# Spontaneous regression of bronchogenic cyst accompanied by pneumonia

**DOI:** 10.1186/s40792-015-0109-2

**Published:** 2015-10-17

**Authors:** Naoya Himuro, Takao Minakata, Yutaka Oshima, Daisuke Kataoka, Shigeru Yamamoto, Mitsutaka Kadokura

**Affiliations:** Division of Chest Surgery, Department of Surgery, Showa University School of Medicine, 1-5-8 Hatanodai, Shinagawa-ku, Tokyo Japan

**Keywords:** Spontaneous regression, Bronchogenic cyst, Pneumonia, Mediastinal tumor

## Abstract

Bronchogenic cysts arise from abnormal budding of the ventral diverticulum of the foregut or tracheobronchial tree during embryogenesis, are the most common cystic masses in the mediastinum, and are generally asymptomatic. A spontaneous regression in a mediastinal bronchogenic cyst (MBC) with pneumonia is rare. A 30-year-old male had a tumor shadow in the middle mediastinum. When he visited our hospital, he had a mild fever with coughing and sputum. A chest computed tomography (CT) scan showed a decrease in the tumor size and the existence of right pneumonia. MBC may be involved in the etiology of pneumonia; therefore, bronchogenic cysts need to be resected as soon as possible.

## Background

A bronchogenic cyst is a relatively rare condition caused by abnormal budding, isolation, and migration of the intrathoracic airway during the fetal period. Cystic lesions account for 14 % of mediastinal tumors, among which bronchogenic cysts are commonly observed. Although most bronchogenic cysts in the mediastinum are generally asymptomatic, some cases may be accompanied by symptoms such as fever, coughing, chest pain, or dyspnea. A decrease in the tumor size of a mediastinal bronchogenic cyst (MBC) accompanied by pneumonia is very rare.

## Case presentation

**Fig. 1:**
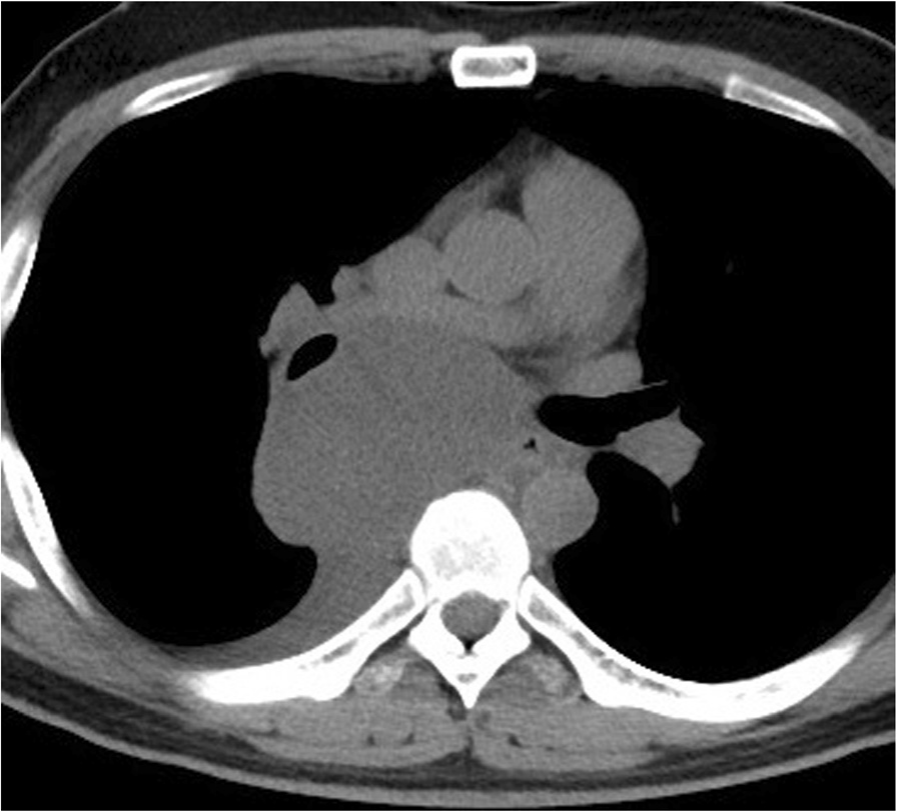
Initial chest computed tomography scan showing the mediastinal tumor measuring approximately 60 mm in length with right pleural effusion

A 30-year-old male visited a local doctor due to the onset of coughing with sputum. He had not previously had any health problems. A chest roentgenogram revealed a tumor in the right hilum. A tumor measuring approximately 60 mm in length was detected in the middle mediastinum by plain chest computed tomography (CT), with a small amount of pleural effusion inside the right thoracic cavity (Fig 1). Magnetic resonance imaging (MRI) revealed a cystic mass. Therefore, the patient was diagnosed with a middle mediastinal tumor and was referred to our hospital. Although he had a mild fever with coughing with sputum, no other symptoms were observed at the initial visit to our hospital. Laboratory tests showed a mildly elevated inflammatory response. Sputum cultures were negative for tuberculosis, fungi, and other bacteria, and the results of cytological studies were unremarkable.

**Fig. 2:**
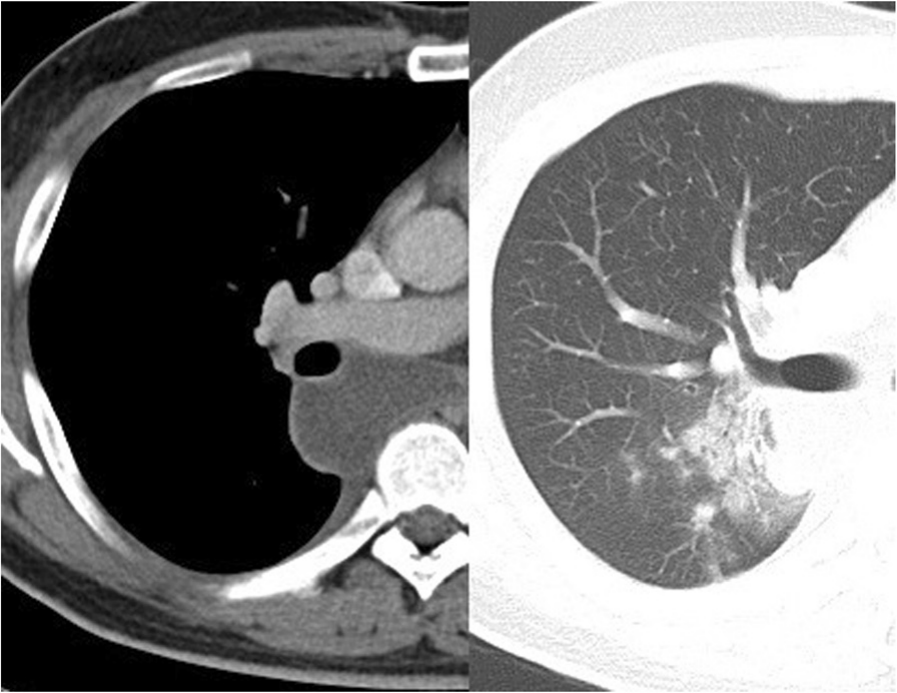
Enhanced computed tomography scan at our hospital showing a reduction in the tumor and right intrapulmonary consolidation

A chest-enhanced CT scan taken at our hospital showed spontaneous regression in the tumor size and right intrapulmonary consolidation, which came in contact with the tumor (Fig 2). Therefore, we commenced the administration of antibiotics. The radiographic intrapulmonary consolidation subsequently disappeared, C-reactive protein (CRP) became negative, and pyrexia improved. Bronchofiberscopic findings revealed no endobronchial abnormalities. Since the infection had improved, mediastinal tumor resection was planned.

**Fig. 3:**
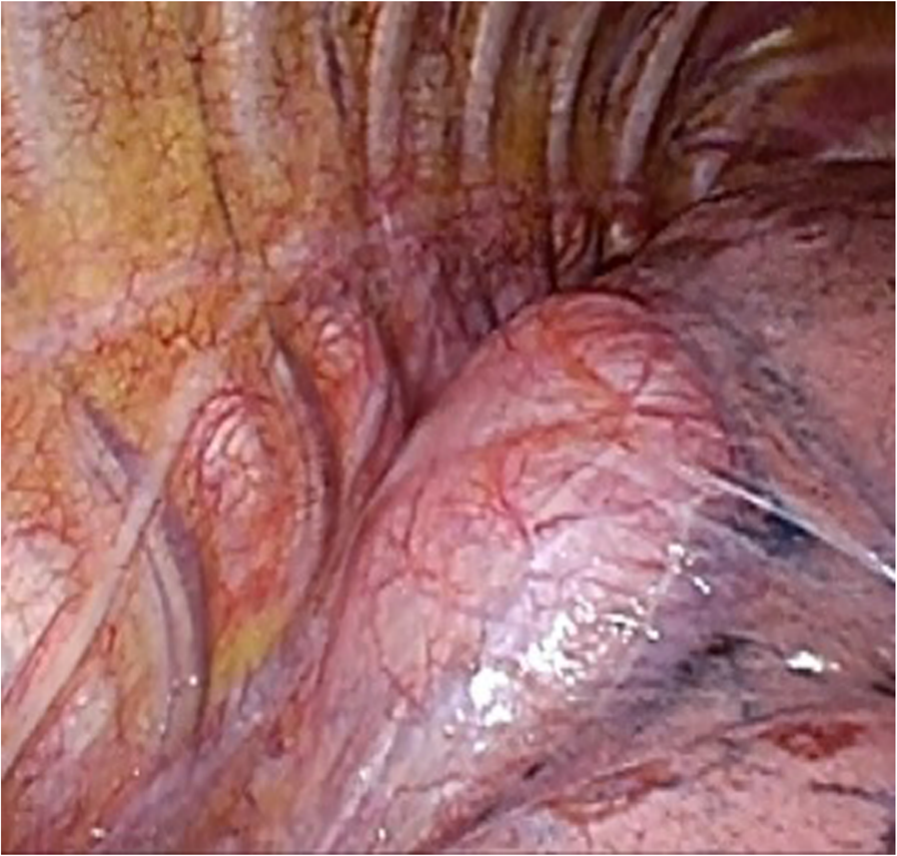
We performed tumor resection by a video-assisted thoracic surgery approach. There was strong fibrous adhesion between the tumor and right lung

We performed tumor resection using the VATS approach. Regarding findings in the thoracic cavity, fibrous adhesion was detected between the tumor and right lung (Fig 3). After the liquid contents of the tumor had been removed, the wall of the cyst was resected as much as possible. There was no bacterium in the liquid contents. Since part of the tumor strongly adhered to the bronchial wall, complete resection of the cyst wall was difficult. Therefore, the wall of the intracystic cavity was cauterized with an electronic knife.

**Fig. 4:**
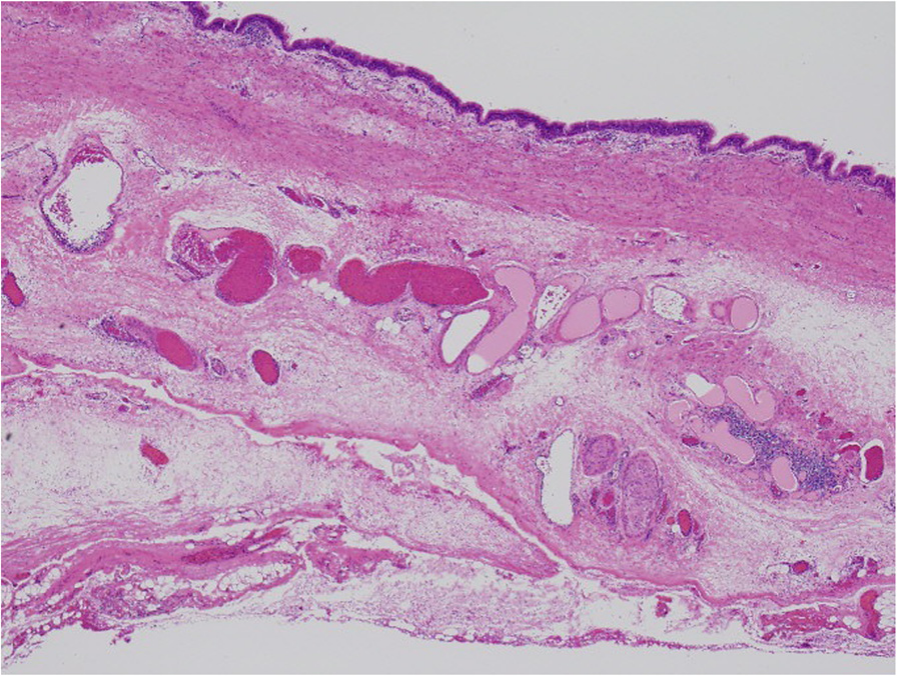
The cyst wall was lined by ciliated columnar epithelia, and the wall contained areas of bronchial glands and smooth muscle cells (hematoxylin-eosin stain ×100)

**Fig. 5:**
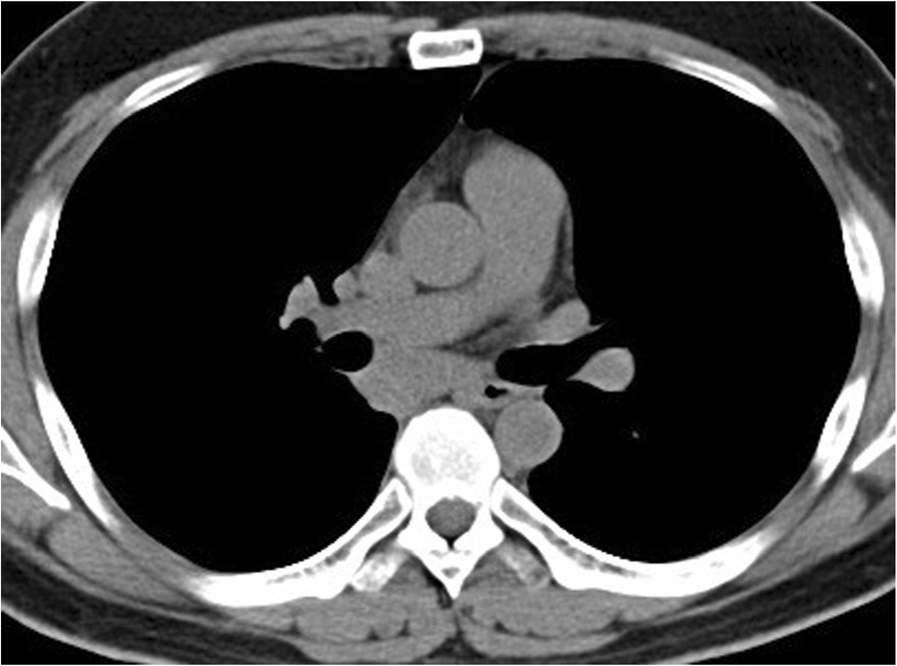
Computed tomography scan after 14 months postoperatively showing no recurrence of MBC

Histopathological findings revealed that the cyst wall was mainly composed of bronchial glands and smooth muscle tissues, with hematoxylin and eosin staining showing that it was lined by ciliated columnar epithelia (Fig 4). Therefore, the tumor was diagnosed as MBC. The patient has been doing well for 14 months postoperatively (Fig 5).

### Discussion

The clinical course of this patient showed that MBC may cause pneumonia with spontaneous reductions. Bronchogenic cysts appear in the respiratory primordium during embryonic development of the foregut and have a relatively high incidence among cystic diseases of the middle to posterior mediastinum. Bronchogenic cysts have been classified into intrapulmonary and mediastinal types, with the intrapulmonary type being more common than the mediastinal type in young patients [1]. Many MBCs are detected without symptoms. Previous studies suggested that MBC was associated with symptoms such as fever, coughing, chest pain, and dyspnea, all of which are caused by intratumoral infections or the compression of adjacent organs [1, 2]; however, a decrease in the tumor size of MBC accompanied by pneumonia is very rare.

The cause of the decrease in the tumor size of MBC with pneumonia may be weak communication between the tumor and tracheobronchial airway. We have only found three cases of pneumonia due to the rupture of bronchogenic cysts into the airway. Of these, the case from Korea was caused by fine needle aspiration biopsy via a bronchofiberscope. In the case from India, viscous cystic contents clogged up the airway due to rupture, leading to respiratory arrest [3–5]. In our case, there was no evidence of rupture of the bronchogenic cyst into the tracheobronchial airway such as air bubbles inside, or communication with the tracheobronchial airway. However, inflammatory changes in the cyst and surrounding organs without communication with the tracheobronchial airway have been shown to account for 1.5 % of bronchogenic cysts [6].

The diagnostic rate of bronchogenic cysts based on CT findings is approximately 60 %, with MRI often being used in combination with CT for a definitive diagnosis [1, 2]. Since imaging findings are difficult to interpret due to intracystic contents, making a definite diagnosis prior to surgery is challenging [2]. Moreover, although endobronchial ultrasound-guided transbronchial needle aspiration (EBUS-TBNA) has been considered as a diagnostic method, tracheobronchial rupture has been reported; therefore, its safety has not yet been established [5]. Surgical resection is also difficult in such cases due to the strong adhesion of such cysts to adjacent organs [7].

In addition to surgical resection, other methods, such as removing intracystic contents with a bronchofiberscope and infusing ethanol, have been reported as treatments for MBC. However, the safety and radical curability of these methods have not yet been demonstrated. Therefore, surgical resection is currently considered preferable and needs to be performed as soon as possible because MBC may lead to pneumonia.

Complete resection is recommended in order to avoid any recurrence due to incomplete resection [8]. However, the risk of injury to adjacent organs increases with strong adhesion to the surrounding area. Previous studies demonstrated that cauterization or dull curettage of the remaining cyst wall effectively prevented recurrence and malignant transformation when complete resection was difficult [1]. In our case, the risk of injury to the bronchus and lungs was high due to strong adhesion, and, thus, as much of the cyst wall as possible was resected. The wall of the intracystic cavity was then cauterized using an electronic knife.

Although the recurrence of bronchogenic cysts is rare, it has been reported as late as 15 years after surgical treatments [8].

## Conclusions

We herein reported the spontaneous regression of MBC accompanied by pneumonia. Although this condition is relatively rare, it may result in a fatal outcome. Therefore, surgical resection needs to be performed as soon as possible, and such cases followed-up for a long period after surgical treatments.

## Consent

Written informed consent was obtained from the patient for the publication of this case report and any accompanying images.

## Abbreviations

CRP: C-reactive protein
CT: computed tomography
EBUS-TBNA: endobronchial ultrasound-guided transbronchial needle aspiration
MBC: mediastinal bronchogenic cyst
MRI: magnetic resonance imaging
VATS: video-assisted thoracic surgery
